# Minding the child’s mind: parental reflective functioning as a buffer for child distress in displaced families at war zones

**DOI:** 10.3389/fpsyg.2025.1684898

**Published:** 2025-11-24

**Authors:** Yael Enav, Yael Mayer

**Affiliations:** School of Therapy, Counseling, and Human Development, University of Haifa, Haifa, Israel

**Keywords:** stress, parenting, children, war, displacement, parent–child relationship

## Abstract

**Background:**

Despite being an exceptionally vulnerable group, internally displaced children are underrepresented in the literature. Parents are instrumental in providing support to these children.

**Objective:**

The aim of this study is to investigate the moderating role of perceived parental reflective functioning (PRF) in the relationship between parental war exposure, parental stress, and parent-rated child distress.

**Participants and method:**

Ninety-six internally displaced Israeli families with young children ages 3–6 were involved. Data was gathered online through online questionnaires using Qualtrics. Parents completed self-report measures assessing their exposure to war-related events, levels of stress and depression (Depression and Anxiety Stress Scale), child distress (Child Stress Scale), and PRF (Parental Reflective Functioning Questionnaire).

**Results:**

Parental reflective functioning (PRF) moderated the indirect link between parental war exposure and parental ratings of child distress via parental stress. At low and moderate PRF, parental stress fully mediated this relationship (Moderate: indirect = 0.05, *p* < 0.05, 95% CI [0.01, 0.09]; Low: indirect = 0.09, *p* < 0.05, 95% CI [0.02, 0.17]). At high PRF, the indirect effect was non-significant, and a direct association emerged (*b* = 0.11, *p* = 0.007), suggesting that highly reflective parents may be more directly attuned to children’s emotional responses to war exposure.

**Conclusion:**

These findings highlight the protective role of PRF in war and support the development of PRF-enhancing interventions to promote resilience among young, internally displaced children.

## 1 Introduction

The events that unfolded on October 7th, 2023 in Israel resulted in war and the internal displacement of hundreds of thousands of individuals across the country (Mayer et al., 2024; Peleg and Gendelman, 2023). Family units are especially vulnerable to the negative effects associated with displacement as the loss of resources contributes to the unraveling of the family’s healthy functioning (Eltanamly et al., 2021; Shyroka et al., 2023; Sim et al., 2018). Children may develop new trauma-based behaviors such as temper tantrums or excessive crying (El-Khani et al., 2020) while parents can experience a significant loss of parental efficacy, competence, and parenting sensitivity in response to rapidly changing environmental and familial conditions (Akesson and Sousa, 2020; Eltanamly et al., 2021).

Parents struggle with how to discuss war and displacement with their children (Dalgaard et al., 2019; Joshi and Fayyad, 2015), experience a lower threshold of tolerance amid severe stress (Eltanamly et al., 2021; Gillespie et al., 2022), grow susceptible to symptoms of PTSD and depression (Eltanamly et al., 2021), and unintentionally engage in harsher parenting practices due to the uncertainty and violence associated with wartime (El-Khani et al., 2017; Ponguta et al., 2020; Zanbar et al., 2023). For children, violent experiences associated with war and displacement can result in symptoms of PTSD (Diamond et al., 2010) and impact development across emotional, psychological, and cognitive domains (Blackmore et al., 2020; Erdemir, 2022b; Gillespie et al., 2022). Symptoms of anxiety and depression, withdrawal, and somatic complaints are common among young children exposed to war and displacement (Erdemir, 2022b).

Parental reflective functioning (PRF) has been identified as a protective factor among displaced parents and children in war. PRF refers to a parent’s capacity to understand their own, and their child’s, mental states – such as thoughts, emotions, and intentions – and recognize how these mental states impact their child’s behavior and the parent–child relationship (Luyten et al., 2017; Schultheis et al., 2019). PRF is imperative to the healthy development of children, the parent–child relationship, and the creation of a secure attachment style (León and Olhaberry, 2020). Effective PRF contributes to the development of children’s emotion regulation skills, self-control, and RF abilities while also improving emotional availability and buffering against the effects of stress among parents (Schultheis et al., 2019). Conversely, low levels of PRF are associated with emotion dysregulation, anxiety, depression, heightened stress, hopelessness, and a tendency toward more hostile or neglectful parenting behaviors (Akesson and Sousa, 2020; Decarli et al., 2023; Moreira and Fonseca, 2022).

Interventions that target parenting can buffer the effects of war and displacement on parenting skills and, by extension, buffer the effects of negative parenting on children’s development (Joshi and Fayyad, 2015; Lavi et al., 2016; Ponguta et al., 2020). Recent research underscores the importance of age in moderating the effects of war exposure. Rachamim et al. (2025) found that the associations between maternal PTSD, continuous traumatic stress, and child PTSD symptoms were strongest among preschool-aged children in comparison to their older peers. The majority of pre-existing interventions are slim and focus on different age groups (Kristen et al., 2024) or primarily focus on parenting education rather than targeting parents’ mental health directly (Kaptan et al., 2021).

The current study aims to contribute to this growing body of work by specifically examining PRF among displaced families with preschool-aged children (ages 3–6), a population at heightened risk and deserving of focused attention. We investigate the mediating role of PRF among direct exposure to war events, parental stress, and child distress. Results will provide essential insights into how enhancing PRF can alleviate the adverse effects of war and displacement on both parents and children. We hypothesize that higher levels of PRF will be associated with reduced parental stress and lower child distress. In the presence of varying levels of PRF, the moderation through parental stress on child distress will be different. Specifically, we hypothesize increased PRF will buffer against the negative effects of war exposure on parental stress and subsequently reduce children’s distress. Owing to the transient and sensitive nature of displaced families and the profound emotional exhaustion associated with surviving during wartime (Ponguta et al., 2020), it was difficult to recruit a large sample of participants for our study. The reality of war and displacement dictates parents must prioritize survival over participation in research studies in order to best protect their family’s safety and interests.

## 2 Methods

The cross-sectional design of this study examines the moderating role of PRF among parental war exposure and child distress via parental stress. Through the use of questionnaires, parent and child variables like PRF, depression and anxiety, child stress, and exposure to war were obtained. General, non-clinical samples of displaced populations were sampled across the country of Israel. The study ran from January 2024–June 2024 and was conducted across the country in different cities that absorbed displaced families.

### 2.1 Participants

Participants in this study were parents of young children (ages 3–6) who had been displaced from their homes in Israel due to the outbreak of war on October 7, 2023 (see Table 1). Families were eligible for inclusion if (1) they had experienced displacement from either southern or northern Israel as a direct result of the conflict, and (2) had at least one child between the ages of 3 and 6. Displacement was defined as being forced to relocate from their primary residence due to physical danger, evacuation orders, or destruction of property related to war activity. A convenience sampling method was used to recruit participants from various shelters and communities.

**Table 1:** Descriptive statistics of respondents’ background characteristics.

Characteristics	Count	Percent
All	96	100.0
Parent’s gender		
Male	12	12.5
Female	84	87.5
Child’s gender		
Male	56	58.3
Female	40	41.7
Income categories		
Below average	31	32.3
Average	22	22.9
Above average	43	44.8
Residential location		
North	49	51.0
South	47	49.0
Parent education		
High school	18	18.8
BA	45	46.9
MA+	33	34.4
	Mean	SD
Child age	4.69	1.14
Parent age	39.00	4.53

***p < 0.001, **p < 0.01, *p < 0.05, ~ *p* < 0.10.

Compensation was not offered to participants and participation in the research was free. To connect with displaced families, our research team collaborated with community centers in their areas of displacement. Participants were motivated to complete measures as they were registered for a free intervention workshop, Home Within the Heart (n.d.). This workshop is a group-based, parent–child dyadic intervention aimed toward boosting the parent–child relationship of those living in wartime. The program consists of four 1-h structured group sessions involving both parents and children, emphasizing emotional attunement, reflective parenting, play-based interaction, and co-regulation strategies. Data gathered from participants was prior to their participation in the program.

Our sample comprised 97 parents and one respondent was dropped due to unavailability of data resulting in *N* = 96. The majority were female respondents (87.5%), whereas the children’s gender affiliation was more balanced (58.3% boys, 41.7% girls). These respondents were evacuated from either the south (49.0%) or the north (51.0%) in Israel at an approximately equal ratio. The children’s mean age was 4.69 (SD = 1.14), and the parents’ mean age was 39.00 (SD = 4.53). The majority of participants demonstrated average or high income and education levels. This is noteworthy as parents of higher education and financial status tend to demonstrate more sensitive parenting skills (Erdemir, 2022a). However, due to the convenience sampling method and recruitment from support sites, the sample may not fully represent the broader population of displaced families.

### 2.2 Procedure

Parents completed questionnaires concerning their own and their child’s mental states prior to the Home Within the Heart intervention. Parents were contacted over the phone and research assistants explained the study. The questionnaires, as facilitated by the Qualtrics online survey program, were sent to parents once they agreed to the study. Informed consent was obtained from all participants, ensuring participants were fully aware of the study’s aims and procedures. Participant confidentiality was maintained by assigning unique identifiers and securely storing data.

In order to reduce bias, we implemented validated measures in the participants’ native language in order to promote transparency and reduce the cognitive load on displaced parents. Participants were gathered through reaching out to multiple communities across the country to promote the diversity of geographic location of displaced families. Additionally, demographic variables such as location of residence and gender were included as control variables in the current study’s analysis. Lastly, a moderated mediation analysis was employed to allow for a more complex interpretation of the data.

### 2.3 Measures

All measures were adapted to participants’ native language of Hebrew and have been validated in previous studies.

#### 2.3.1 Demographic questionnaire

Basic demographic information such as age, gender, marital status, education level, financial status, and religiosity were gathered regarding the parents’ profiles. The latter was collected as all participants were Jewish Israelis. Questions regarding displacement such as identifying the family’s original location of residence, new location, and how long they have been displaced were also employed. Child demographic factors such as age, gender, birth order, and general health information were assessed as well.

#### 2.3.2 Exposure to War-Related Events Questionnaire

This questionnaire was adapted from the Political Life Events Questionnaire (PLE; Slone et al., 1997) and included items related to exposure to war-related events. These events encompassed individual experiences or those of close associates regarding violence, abduction, evacuation, exposure to air raid sirens, and economic and familial challenges. Participants rated items on a 5-point Likert scale ranging from 1 = “I was not exposed” to 5 = “I was exposed and it was extremely stressful.” Further, this scale demonstrates high test–retest reliability at r = 0.87 and appropriate cross-cultural application (Lavi and Slone, 2012; Slone et al., 2008). The final scale was a count across binary (zero if not, one otherwise) responses to a list of stressful war events. Overall, the mean value of these stressful events was 2.81 (SD = 1.77). Exposure was found to be higher among families from the south in comparison to those from the north (south: Mean = 3.70. SD = 1.50; north: Mean = 2.02, SD = 1.50; t(96) = −5.30, *p* < 0.001). Internal consistency was unassessed as, drawing on previous research working with the PLE, consistency in war exposure is not expected (Lavi and Slone, 2012; Slone et al., 2008).

#### 2.3.3 Depression and Anxiety Stress Scale (DASS-21)

The DASS-21 is a 21-item self-report instrument designed to assess anxiety, stress, and depression (Lovibond and Lovibond, 1995). It includes items such as “I found it difficult to relax,” and “I felt scared without any good reason.” Employing the Hebrew version of the scale (Palgi et al., 2019), participants responded to each item on a 4-point Likert scale, ranging from 1 = “did not apply to me at all” to 4 = “applied to me very much.” In the current study, the DASS-21 demonstrated excellent internal consistency as evidenced by an alpha coefficient of 0.95, Mean = 1.75, SD = 0.53. No location difference was found (*t* = −0.89, *p* = 0.374).

#### 2.3.4 Child Stress Scale (SRCL)

Stress reactions were assessed using the Stress Reaction Checklist (SRCL; Sadeh et al., 2008). The SRCL comprises 15 items which includes fear of, or strong reactions to noise, fear of separation, passivity and disinterest in play, and nightmares. Parents rated the extent to which their child exhibited each behavior on a scale of 1 = “not at all” to 3 = “severe” and indicated whether the behavior existed before the war. The SRCL demonstrated good internal reliability with a Cronbach’s alpha of 0.79, Mean = 1.84, SD = 0.43. No difference was found among those displaced from the north and those displaced from the south (*t* = 1.59, *p* = 0.116). This questionnaire was translated from English into Hebrew as part of a rigorous process to ensure accuracy and cultural relevance for our sample population. Reverse translation to English was then employed to ensure the consistency and reliability of the Hebrew translation of the SRCL. Any discrepancies that may have arisen within the Hebrew translation were reviewed collaboratively by the research team in order to maintain the integrity of the questionnaire’s original English content.

#### 2.3.5 Parental Reflective Functioning Questionnaire (PRFQ)

The Parental Reflective Functioning Questionnaire (PRFQ; Luyten et al., 2017) was utilized to assess perceived parental reflective functioning as self-reported by participants. The Hebrew version was employed (Shai et al., 2017) and we specifically focused on the Interest and Curiosity subscale, as scores in this subscale are related to parental emotional availability (Luyten et al., 2017). An example item from this subscale is, “I am often curious to find out how my child feels.” Parents rated each item on a Likert scale ranging from 1 = “Strongly Disagree” to 7 = “Strongly Agree.” The responses *for each item were averaged to produce a PRF score, with higher scores indicating greater self-reported* reflective functioning skills in parents. The PRFQ has demonstrated good internal consistency, evidenced by an alpha coefficient of 0.88, Mean = 5.97, SD = 0.99. No location difference was found (*t* = −0.21, *p* = 0.834).

### 2.4 Data analysis

Data gathered was analyzed using SPSS (version 27) and the PROCESS macro for mediation and moderation analysis (Hayes, 2018). Descriptive statistics were calculated for all control variables: child’s age, parent’s gender, and northern vs. southern residence (see Table 1). Note that there were no missing values and no sensitivity analysis was performed. Time elapsed since evacuation was not included as a separate correlation variable due to the uniformity in timing; most families were displaced simultaneously following the October 7th attack, with interviews conducted within a relatively narrow window (between 4 and 6 months post-evacuation). As a result, variation in displacement timing was minimal and closely tied to the interview date. To capture meaningful differences in displacement status, we used residential location (north vs. south) as a proxy: families from the north were generally able to return home, while families from the South remained displaced at the time of data collection. This binary variable was included as a control in the models to reflect differential experiences of ongoing displacement.

**Figure 1:**
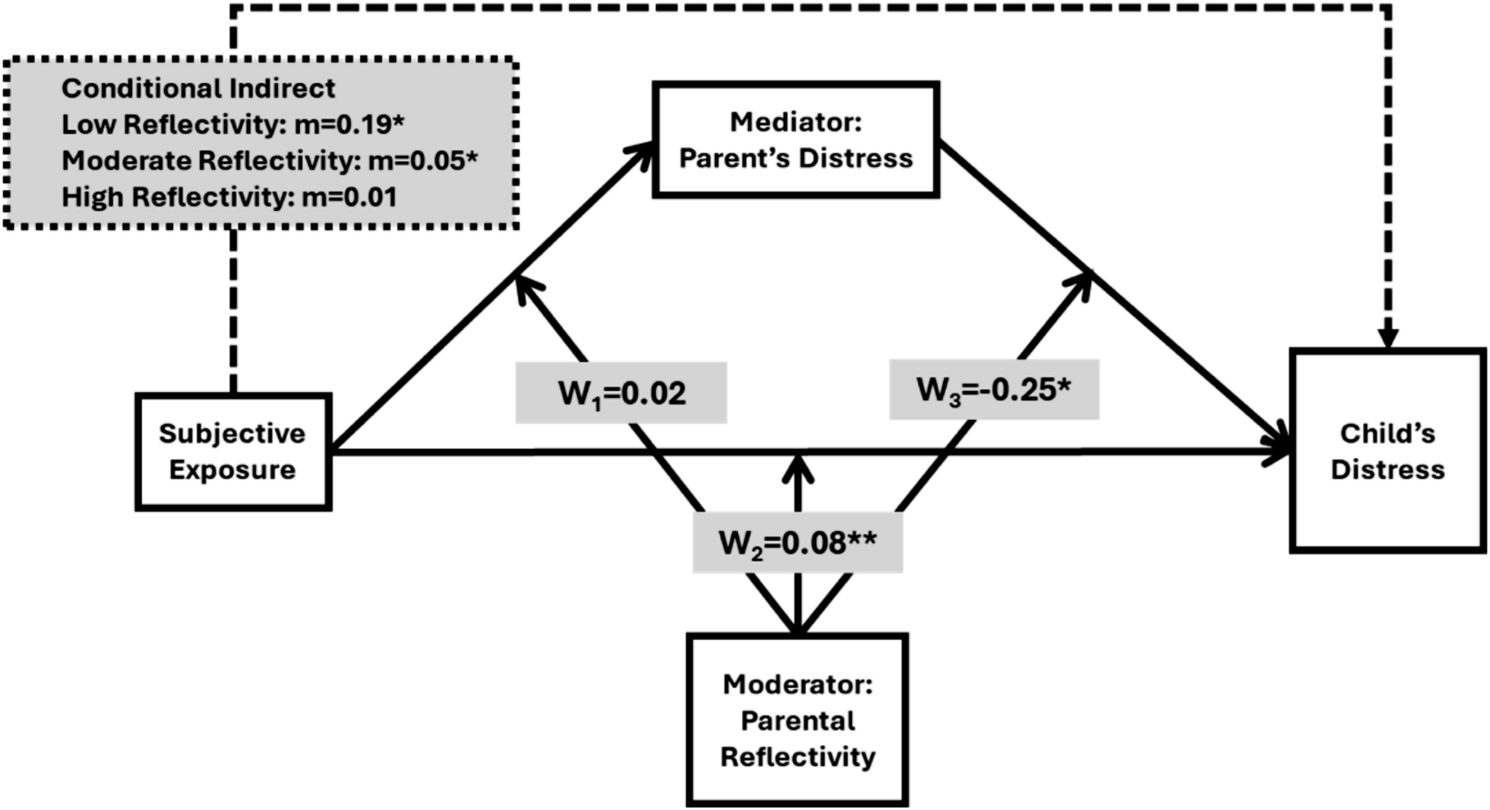
Moderated mediation model of the association between exposure to war events and child distress, mediated by parental stress and moderated by parental reflective functioning (PRF). Arrows indicate direct and indirect paths, with moderation effects depicted on each path.

The hypotheses were tested using a moderated mediation model, where PRF is the moderator between direct exposure to war events and child distress, and parental stress is the mediator on all mediation slopes; that is, from IV to M, from M to DV, and from IV to DV. Regression analyses were performed to assess direct effects of exposure and reflectivity on parents’ distress and children’s distress reaction, followed by the effect of an interaction between exposure and reflectivity. A final application of the moderated mediation model (PROCESS, Model 59) was performed to evaluate the parents’ distress mediation role in explaining exposure effect on children’s distress reaction, which was conditioned by parental reflectivity (see Figure 1). Note, however, that by mediation we mean indirect effect rather than any causal effect, except that exposure was prior to stress and distress. A more detailed explanation of the model is provided below. A preliminary power analysis was performed using two independent results for the two regression equations for which we received power (1 − *β*) of 0.88 for the first, and 0.92 for the second, given alpha of 0.05, moderate to high effect size of 0.20 based on the actual effects, and sample size of 95. This implied a power of 0.809, above the accepted threshold.

### 2.5 Ethical considerations

The study adhered to ethical guidelines as outlined by the Institutional Review Board of the University of Haifa (approval number 003/24) and in accordance with the Helsinki Declaration of Human Rights (World Medical Association, 2013). Informed consent was obtained from all participants, ensuring they were fully aware of the study’s aims and procedures. Participant confidentiality was maintained by assigning unique identifiers and securely storing data. Participants were told they are free to stop at any time during the study. Usable data was collected from 96 parents and presented in this manuscript.

### 2.6 Data availability

Data will be made available by the corresponding author within a reasonable timeframe upon request.

## 3 Results

Our core inquiry guiding this research was how subjective, direct parental exposure to war events was associated with children’s emotional distress. To answer this, we developed a moderated mediation model in which parental response to war exposure by means of parental stress and depression was a mediator of the child’s distress. That is, in part or even completely, the war exposure-distress association was expected to be explained by the presence of parents’ distress. However, this mediation effect was controlled by PRF. In other words, we expected the mediation effect to vary with respect to varying values of PRF. We adopted the PROCESS procedure (Hayes, 2018) to test these moderated mediation (see previous emphasis on indirect effect) relations between war exposure and child’s distress. This allowed us to answer a more general question as to the transfer of parental emotional response onto their children during stressful situations. An illustration with further estimates of the complete moderated mediation model is shown in Figure 1 wherein WI, W2, W3 are moderating effects on slope a, slope b, and slope c, respectively. In regression terms, W1 is the interaction between war exposure and PRF on parental distress, W2 is the interaction between war exposure and PRF on the child’s distress, and W3 is the interaction between parents’ PRF and the mediator on child’s distress. By the moderating effect we emphasize that each of the three slopes which were involved in assessing the mediation varies with respect to varying values of parental reflectivity. This model was tested within a regression framework that included the child’s age, parent’s gender, and residential location (south versus north) as controls on the outcome of the child’s distress. Residential location was explored as, at the time of data collection, those in the south were directly attacked by Hamas while those in the north were unsure if Hezbollah would join the attack, meaning that the short-term future of displaced families from the north was perceived as less clear.

### 3.1 Moderated mediation (indirect) model results

Table 2 shows modeling results which include direct and indirect effects as well as the interaction effects. Two regression models were performed to generate the indirect effects: 1. from independent exposure to the mediating parents’ distress as measured by the DAS, and 2. from the independent and the mediator variables to the outcome of the child’s distress. Child’s age, parent’s gender, and location of residence did not yield an effect on parental distress but subjective war exposure did (*β* = 0.48, *p* < 0.001); or greater levels of war exposure was associated with stronger parental distress. These controls, however, showed a significant effect on the child’s distress (Child’s age: *β* = −0.22, *p* < 0.05; Residence: *β* = −0.32, *p* < 0.01; Exposure: *β* = 0.28, *p* < 0.05; DAS: *β* = 0.32, *p* < 0.01). The interaction effects W2 and W3 were found to be significant (*β* = 0.08, *p* < 0.01; *β* = −0.25, *p* < 0.05; respectively), which means that the indirect effect varied with respect to PRF values.

**Table 2:** The effects of subjective war exposure on the child’s stress reaction by DAS.

Characteristics	Parental depression and stress	Child’s stress reaction
Standardized main effects		
Child’s age	−0.17	−0.22*
Parent’s gender	0.06	0.06
Residence: south vs. north	−0.17	−0.32**
Subjective exposure	0.48***	0.28*
Parental reflective functioning PRF	0.02	0.06
DAS	–	0.32**
*F, R^2^*	5.25***, 0.23	4.19***, 0.22
Unstandardized interaction results		
Subjective exposure × Parental reflective functioning	−0.02 (0.03)	0.08**, (0.03)
DAS × Parental reflective functioning	–	−0.25*, (0.11)
*ΔF, ΔR^2^*	0.35, 0.003	4.32*, 0.07
Unstandardized conditioned indirect effects (mediation) of subjective exposure		
Low reflective	–	0.09* (0.04) [0.02, 0.17]
Medium reflective	–	0.05* (0.02) [0.01, 0.09]
High reflective	–	0.01 (0.03) [−0.04, 0.06]

*N* = 96; ***p < 0.001, **p < 0.01, *p < 0.05. Standard error of the estimate in parentheses; 95% confidence interval of the estimates in squared brackets based on bootstrap resampling technique (number of repeats = 1,000).

The indirect effect of parents’ subjective war exposure on their child’s distress is shown in three different conditional values: low, moderate, and high levels of PRF. In the presence of high levels of PRF (mean+1SD), we found the mediation effect to be insignificant (indirect = 0.1, *p* < 0.05). In contrast, in the presence of moderate and low levels of PRF the indirect effect was found to be statistically significant at *p* < 0.05 (Moderate PRF: indirec = 0.05, *p* < 0.05, 95%CI[0.01,0.09]; Low PRF: indirect = 0.09, *p* < 0.05, 95%CI[0.02,0.17]). These findings are shown in Figure 1. However, based on W2 decomposition, the direct effect of exposure on the child’s distress appeared significant only when PRF was at high levels (*b* = 0.11, *p* = 0.007, 95%CI[0.03,0.18]). This means that the conditional indirect effects at the presence of low and moderate PRF were complete. In other words, they were a substitute rather than complementary to the effect of exposure on the child’s distress, Namely, when PRF was low, parental DAS negatively mediated the effect of exposure on child’s distress. When PRF was high, parental DAS was ineffective, though the expected effect of exposure on distress was positive.

Since the direct effect of war exposure on the child’s distress was insignificant, we define the two indirect effects at those low and moderate values of PRF as complete rather than partial. In other words, for those parents with low or moderate PRF, the effect of war exposure on the child’s distress could be explained via the mediation of parents’ distress. In contrast, the positive direct association between war exposure and the child’s distress showed no complementary indirect effect via DAS if the level of PRF was high. In this case, if PRF was present in higher values, the exposure effect on the child’s distress was positive but decreased in value in relation to higher levels of PRF.

## 4 Discussion

The results of this study provide crucial insights into the dynamics between direct exposure to war events, parental stress, and parent-rated child distress, emphasizing the moderating role of parental reflective functioning. Our findings suggest that parental exposure to war events has an indirect effect on child distress as mediated by parental stress and depression. Notably, this effect is insignificant when PRF levels are high, but it becomes significant when PRF levels are medium or low. This suggests that high PRF can mitigate the adverse effects of war exposure on children in displaced families. These findings highlight the potential relevance of PRF in supporting parent and child functioning during wartime.

Our findings align with previous studies that highlight the benefits of parental reflective functioning in fostering positive parent–child interactions (Borelli et al., 2021). Parents with high levels of PRF demonstrated a greater ability to provide emotional support to their children, even in the face of significant stress. This emotional availability acted as a protective factor, helping to buffer children from the adverse psychological effects of parental distress. These findings are consistent with previous research that has demonstrated a strong association between high PRF, effective parenting practices, and better developmental and emotional outcomes for children (Decarli et al., 2023; Enav et al., 2019; Schultheis et al., 2019).

However, it should be noted that the presence of a strong parent–child relationship also boosts PRF (Enav and Mayer, 2025). Therefore, PRF should be viewed as a fluid variable that can be influenced by children in addition to parents themselves. Although this study employed the PRFQ, a self-report measure that is susceptible to bias, it captures parents’ subjective representations of their reflective stance rather than observed reflective behaviors. Therefore, links between PRFQ-assessed reflective functioning and parent–child relationship quality should be interpreted as suggestive rather than definitive. Future studies should integrate interview-based (e.g., the Parent Developmental Interview) or observational methods to assess reflective functioning as enacted in real interactions. Further, existing PRF-focused programs such as *Minding the Baby* (Slade et al., 2005) or *Thinking Emotions* (Enav et al., 2019) primarily evaluate interview-based or observational RF, underscoring the need to align measurement tools with intervention mechanisms.

By capturing parents’ reflective functioning during an ongoing and highly acute trauma context, this study offers rare insight into parental resilience under real-time stress exposure, a context in which reflective capacities are typically most compromised. As expected and consistent with previous studies (Akesson and Sousa, 2020; Eltanamly et al., 2021), exposure to war was found to have a direct association with increased parental stress, regardless of PRF level. Traumatic events related to war, along with the subsequent loss of resources and stability, significantly impacted parents’ mental health. These findings highlight the critical need for adequate support and resources for displaced families to help them navigate the challenges posed by war (El-Khani et al., 2020). Furthermore, a direct association was observed between parental stress and parent-rated child distress. When parents struggle to effectively manage their own stress, their ability to provide the necessary emotional support to their children is compromised. This finding aligns with previous research (Eltanamly et al., 2021; Gillespie et al., 2022; Zanbar et al., 2023). Importantly, this association is moderated by PRF, such that it is significant among parents with low to moderate PRF levels but not among those with high PRF. This supports the protective role of PRF in buffering children from the adverse effects of parental stress.

It is notable that a direct association between parental exposure to war events and parent-rated child stress emerged only among parents with high PRF. In the mediation model, which accounts for parental stress and depression, this direct link is not observed for parents with low or medium PRF. However, when parents experience high exposure to war and have high PRF, their children’s stress levels are significantly affected. This suggests that highly reflective parents may be more attuned to the impact of traumatic events on their children. It is crucial to highlight that the events under discussion are extreme, including displacement, injuries or deaths of family members, and prolonged confinement in bomb shelters.

The finding that mediation via parental stress disappears at high levels of PRF warrants further theoretical consideration. One possible explanation is that high PRF enables parents to maintain emotional availability and attunement to their children’s needs even under extreme stress. When PRF is high, parents are more capable of recognizing and responding to their child’s emotional signals directly, rather than filtering these signals through the lens of their own psychological distress. In statistical terms, this reduces the indirect (mediated) pathway via parental stress as the child’s distress is more directly associated with the objective reality of war exposure as perceived and reflected upon by the parent. In essence, high PRF acts as a buffer that prevents parental stress from becoming the dominant conduit for emotional transmission.

Moreover, PRF may function not only to shield the child from the parent’s stress but also to heighten the parent’s sensitivity to the child’s independent experience of trauma. Reflective parents may be more attuned to subtle signs of child distress that stem directly from the child’s own war-related experiences, such as sensory triggers, separation fears, or play-based trauma reenactment (Feldman, 2019), regardless of the parent’s emotional state. Thus, the direct path between war exposure and child distress becomes more visible in high-PRF families because these parents can detect and acknowledge child distress more clearly. This suggests that PRF does not merely reduce negative emotional contagion; it may transform the entire relational dynamic from one of indirect transmission to one of direct empathic recognition.

Regarding child variables, several factors were found to significantly impact parent-rated child distress. One key determinant was the child’s age, with younger children exhibiting higher levels of distress. This finding aligns with previous research on the vulnerability of younger children to war exposure and their increased risk of anxiety disorders (Halevi et al., 2016). In contrast, older children appear better equipped to cope with stress, likely due to their more developed cognitive and emotional regulation abilities. Additionally, our findings indicate regional differences in subjective war exposure among displaced parents. Those originally from northern Israel reported higher subjective exposure compared to those from the south, despite the latter being directly affected by the October 7th terror attack. This unexpected result may reflect the timing of data collection (January–June 2024), suggesting that trauma in the south may have plateaued, while uncertainty in the north, driven by fears of future conflict, may have contributed to heightened distress.

This has important implications for children as infants and young children are at a heightened risk of developmental disruptions when their parents’ mental health deteriorates (Hazer and Gredebäck, 2023), including delays in motor, language, and socioemotional skills. As these children age they are more likely to experience poor physical and mental health outcomes like anxiety and depression in response to experiencing war and displacement in their childhood (Hanetz-Gamliel and Dollberg, 2025). These children also tend to exhibit poor ER, theory of mind, and empathy skills and each subsequent exposure to war can deteriorate their abilities (Hanetz-Gamliel and Dollberg, 2025; Kara and Selcuk, 2023), underscoring the importance of the parental ability to reflect with their children.

### 4.1 Practical implications

These findings suggest that PRF is a valuable target for interventions aimed at supporting displaced families and can serve as a protective factor against the negative impacts of war exposure (El-Khani et al., 2020). Programs aimed at enhancing PRF skills, stress management, self-efficacy, and emotion regulation are likely to benefit both parents and children (Erdemir, 2021; Erdemir, 2022a; Kristen et al., 2024; Ponguta et al., 2020). However, in cases of high parental war exposure, PRF alone may not be sufficient. It is important to acknowledge that this study was not designed to evaluate the impact of such interventions, nor does it provide evidence for the effectiveness of PRF training in displaced populations. Addressing fundamental needs such as access to medical care, financial stability, and childcare may be more effective in mitigating distress in such cases (Gillespie et al., 2022).

Furthermore, these findings have several implications for policy and intervention efforts. First, programs aimed at displaced families should consider integrating PRF training into early interventions, particularly during the early months following displacement. PRF-enhancing interventions, such as reflective parenting groups, could be adapted into low-resource, culturally sensitive formats for implementation in shelters, schools, or virtual settings. Furthermore, this research points to the value of developing cross-cultural versions of such interventions, especially in global contexts where displacement due to war or natural disaster is increasing. However, research methodology must be considered. While PRFQ-based studies have contributed to understanding parental representations of reflective functioning, empirical evidence connecting changes in PRFQ scores to improvements following reflective interventions remains sparse. Future research should combine self-report and observational measures to determine whether PRFQ-assessed changes reflect genuine shifts in the parental mentalizing process.

From a policy standpoint, funding mechanisms that support relationally focused, trauma-informed care in humanitarian contexts should prioritize parent–child dyadic approaches, particularly for families with young children. Moreover, longitudinal research tracking families across the displacement-resettlement continuum is critical for assessing the lasting benefits of PRF and tailoring supports accordingly. Incorporating PRF into psychosocial assessments used by humanitarian agencies may also help identify families in need of additional support.

### 4.2 Study limitations

While this study offers important insights, several limitations should be noted. First, although the sample size was adequate for the planned analyses, it may not capture the full heterogeneity of displaced families. Recruitment through a free parent–child program constitutes convenience sampling and may introduce selection bias: families who enroll may differ systematically (e.g., motivation, awareness of psychosocial resources, baseline mental health, resilience) from those who do not, limiting generalizability to more isolated or severely traumatized families.

Second, reliance on self-report, particularly parental reports of child distress, may bias estimates when parental perceptions diverge from children’s experiences. Third, the sample was predominantly female; fathers were markedly underrepresented, consistent with broader trends (e.g., Hein et al., 2020), which may reflect role expectations and availability constraints and limits inferences to fathers.

Fourth, parental stress and PRF levels may in part reflect long-standing psychological patterns rather than responses solely to war exposure; combined with the cross-sectional design, this constrains causal inference regarding proposed pathways.

Finally, our child outcomes emphasized parent-reported distress (and adaptive functioning) and did not include positive social outcomes—such as prosocial behavior (helping, sharing, empathy), reciprocity, peer relations—or antisocial behaviors. Future work should incorporate multi-informant (parent/teacher/observer) and multi-method (questionnaires/behavioral tasks) assessments of prosocial and socio-moral development alongside distress to test whether parental stress and PRF are associated with improvements across both negative and positive child outcome.

## Data Availability

The raw data supporting the conclusions of this article will be made available by the authors, without undue reservation.
